# Rebuilding spinal circuit function after spinal cord injury through a patient-specific interneuron precision model

**DOI:** 10.3389/fnins.2026.1745993

**Published:** 2026-06-03

**Authors:** Sasi Kumar Jagadeesan, Ryan Vimukthie Sandarage, Eve C. Tsai

**Affiliations:** 1 Department of Neuroscience, Faculty of Medicine, University of Ottawa, Ottawa, ON, Canada; 2 Neuroscience Program, Ottawa Hospital Research Institute, The Ottawa Hospital, Ottawa, ON, Canada; 3 Department of Neurosurgery, The Ottawa Hospital, Ottawa, ON, Canada

**Keywords:** circuit regeneration, developmental patterning, neural circuit, neuromodulation, spinal cord injury, spinal interneurons

## Abstract

Spinal interneurons constitute the physiological core of spinal circuitry, integrating excitatory and inhibitory inputs to generate the rhythmic patterns that drive locomotor, postural, and autonomic control. Their developmental logic, molecular diversity, and adaptive plasticity make them central determinants of functional recovery after spinal cord injury (SCI). Yet most regenerative strategies continue to emphasize cellular replacement rather than the restoration of the physiological integrity of spinal networks. In this article, we reframe spinal repair as the restoration of interneuron-mediated circuit organization rather than cellular replacement alone. We synthesize current insights into how embryonic patterning programs defined by Sonic Hedgehog (SHH), Wnt, and bone morphogenetic protein (BMP) gradients, refined by Notch and retinoic acid signaling, and consolidated by axon guidance cues, establish interneuron diversity, connectivity, and network symmetry that together encode the logic of motor coordination. SCI disrupts this developmental logic, fragmenting excitatory and inhibitory balance and desynchronizing rhythmic modules, while residual circuits retain latent capacity for resynchronization through plasticity and neuromodulation. Building upon this developmental and physiological continuum, we propose the Patient-Specific Interneuron Precision Model (PIPM), a feedback-informed conceptual framework that links patient-specific biological states, including progenitor competence, morphogen sensitivity, metabolic tone, inflammatory burden, and lesion-specific circuit preservation, to circuit-level function and recovery potential. Frameworks such as the PIPM may help integrate molecular, physiological, and clinical dimensions of recovery, providing a path toward more personalized strategies for treating SCI through restoration of interneuron-mediated network organization.

## 1 Overview

The spinal cord possesses intrinsic rhythmogenic capability that enables patterned motor and autonomic output through central pattern generator (CPG) circuits. However, these networks are not functionally independent of the brain; descending supraspinal systems modulate, stabilize, and selectively gate spinal interneuron ensembles, and their disruption after SCI contributes to abnormal excitability and fragmented motor output (Kiehn, 2016; Sengupta and Bagnall, 2023). This intrinsic capability arises from the organization of spinal circuits, in which diverse excitatory and inhibitory interneuron populations play a central physiological role as components of the CPG (Zholudeva et al., 2021; Gosgnach, 2023). Through their structured synaptic connectivity, these interneurons encode the rhythm, directionality, and coordination of muscle activation across motor pools, sustaining locomotion, posture, and visceral regulation (Grillner and El Manira, 2020; Wilson and Sweeney, 2023). Rather than serving as passive relay elements, interneurons act as the intrinsic coordinators of spinal circuit function, integrating excitatory and inhibitory drives to produce rhythmically organized motor output. Their diversity, spanning multiple progenitor domains and neurotransmitter identities, confers modularity and adaptability, and distinct subtypes mediate left–right alternation, flexor–extensor transitions, and sensory gain control (Ampatzis et al., 2014; Ziskind-Conhaim and Hochman, 2017). Interneurons also establish distributed circuits that dynamically adjust to biomechanical and sensory inputs, facilitating partial functional recovery after injury (Asboth et al., 2018). Despite this central role, most regenerative interventions remain agnostic to interneuron subtype identity or circuit-level organization (Fischer et al., 2024; Kvistad et al., 2024). As a result, structural replacement without restoring the diversity and connectivity of interneurons has yielded inconsistent or incomplete recovery outcomes (Lu et al., 2012).

Conventional transplantation of neural stem or progenitor cells frequently results in incomplete lineage specification and fails to restore the precise excitatory–inhibitory balance required for rhythmic coordination, leading to structural repair that yields limited or incoherent function. These limitations reveal a fundamental conceptual gap, suggesting that replacing neurons does not equate to restoring the physiological integrity of spinal circuits (Tetzlaff et al., 2011; Dulin and Lu, 2014; Ni et al., 2023). Adding further complexity, the biological competence of human neural stem/progenitor cells (NSPCs) is profoundly heterogeneous. This variability directly influences the ability of transplanted or endogenous progenitors to generate appropriate interneuron subtypes and integrate into existing spinal circuits (Ottoboni et al., 2020; Yang et al., 2024; Jagadeesan et al., 2025a). Age, systemic metabolism, chronic inflammation, and prior neurological injury reshape progenitor transcriptional programs and morphogen responsiveness, constraining interneuron differentiation and circuit integration (Elhabbari et al., 2024; Szegedi et al., 2024). Aging induces progenitor quiescence and narrows the spectrum of interneuron lineages produced, while astrocytic and immune reactivity sculpt permissive or restrictive microenvironments that govern interneuron survival and connectivity (Szegedi et al., 2024; Jagadeesan et al., 2025a). These patient-specific variables could likely underlie the inconsistent interneuron patterning and circuit outcomes observed across ostensibly similar regenerative interventions.

At the systems level, several quantitative and conceptual models have been proposed to guide spinal repair, ranging from morphogen-based developmental maps to network simulations of CPG organization. However, most remain generalized and do not account for patient-specific variability in interneuron competence, subtype identity, or network integration (Rybak et al., 2006, 2015). Consequently, regenerative strategies derived from such population-level frameworks often fail to predict or reproduce patient-specific outcomes, highlighting a major conceptual gap between cellular potential and the restoration of functional interneuron circuitry. Frameworks that could integrate patient-specific biological competence, developmental patterning logic, and circuit-level feedback may offer a more tractable path toward precision-guided spinal repair. One such framework is the PIPM, which proposes to reframe spinal regeneration as the restoration of coordinated circuit function rather than structural continuity alone. The model positions interneurons as the core functional organizing units of spinal circuitry and integrates these variables to guide strategies hypothesized to restore rhythmic and coordinated function. Through the integration of cellular state variables and emergent network dynamics, the PIPM offers a conceptual foundation for stratifying regenerative hypotheses and guiding future intervention design across defined interneuron subtypes and circuit motifs.

## 2 Developmental and circuit logic

**Figure 1:**
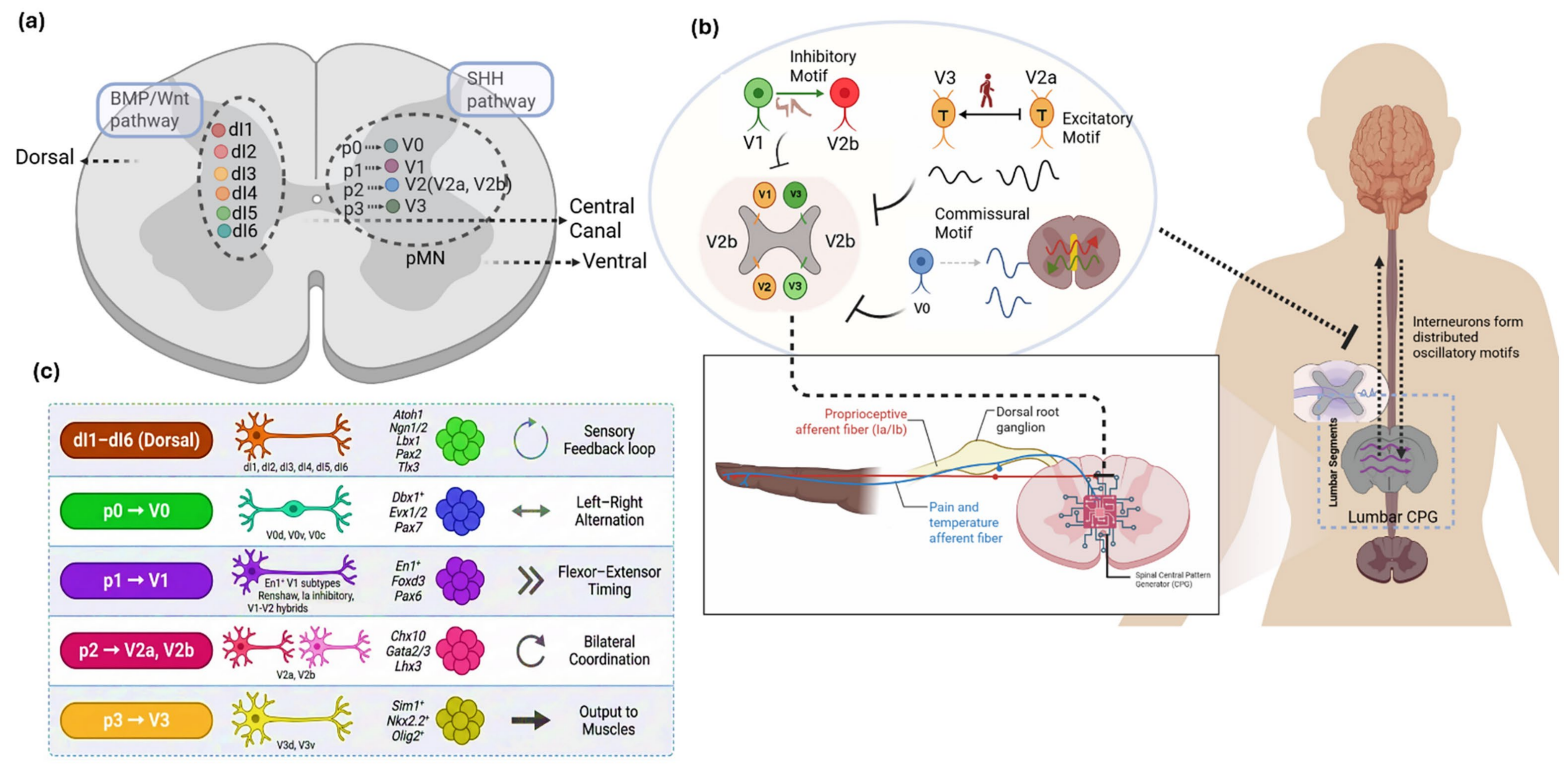
Developmental and functional logic of spinal interneuron diversity and its translational relevance. **(a)** Dorsoventral morphogen gradients, including BMP/Wnt dorsally and SHH ventrally, organize the embryonic spinal cord into discrete progenitor domains (p0–p3, dI1–dI6) that establish spatial and molecular identity through transcriptional cross-repression. **(b)** Ventral interneuron subtypes (V0–V3) and dorsal sensory interneurons assemble into inhibitory, excitatory, commissural, and sensory-feedback motifs that contribute to spinal central pattern generator (CPG) organization. V0 commissural neurons coordinate left–right alternation, V1 inhibitory neurons regulate flexor–extensor timing and reflex inhibition, V2a and V2b populations contribute to excitatory–inhibitory balance, and V3 excitatory commissural neurons support bilateral rhythm and rostrocaudal coordination. Proprioceptive Ia/Ib afferents and nociceptive/temperature afferents provide sensory inputs that interact with interneuron circuits to regulate reflex gain and motor output. **(c)** Domain-specific transcription factor combinations delineate lineage boundaries and contribute to circuit function, while temporal morphogen exposure (SHH, Wnt, BMP, Notch, RA) defines dorsal and ventral bias and developmental competence. The schematic also highlights intra-domain heterogeneity, including V1-associated Renshaw and Ia inhibitory interneuron populations and V3 dorsal/ventral subtypes, emphasizing that progenitor domains represent organizational scaffolds rather than homogeneous functional classes.

Spinal interneurons arise from a spatially ordered developmental program that translates dorsoventral patterning cues into the structural and functional architecture of the mature spinal cord (Vallstedt and Kullander, 2013; Goyal et al., 2020). Gradients of SHH (ventral floor plate) and BMP/Wnt (dorsal roof plate) establish progenitor domains (p0-p3 and dI1-dI6) through graded *Gli* signaling and cross-repressive transcriptional networks involving *Dbx1*, *En1*, *Chx10*, *Lhx3*, and *Sim1* (Figure 1a). Temporal regulation by Notch and retinoic acid signaling further refines lineage competence, while axon guidance systems including Netrins, Slits, Ephrins, and Semaphorins direct projection trajectories that consolidate spatial identity into circuit organization (Francius et al., 2013). The coordinated action of these molecular programs produces a reproducible map of interneuron subtypes whose intrinsic electrophysiological and synaptic properties reflect their developmental origin (Figures 1b,c).

Each class contributes a discrete physiological role within the CPG network. V0 neurons derived from *Dbx1* progenitors project commissurally to coordinate left–right alternation, whereas V1 interneurons defined by *En1* expression regulate flexor–extensor transitions and temporal precision (Francius et al., 2013; Chapman et al., 2025). V2 interneurons diverge into excitatory V2a (*Chx10*^+^) and inhibitory V2b (*Gata2/3*^+^) subtypes that balance excitation and inhibition within motor pools, while V3 neurons marked by *Sim1* expression maintain bilateral rhythm and robustness. Dorsal interneurons (dI1–dI6) integrate proprioceptive and cutaneous inputs to couple sensory feedback to motor output (Lundfald et al., 2007; Chopek et al., 2018).

Muscle spindle afferents, group Ia fibers signaling muscle length and velocity, and group Ib fibers from Golgi tendon organs signaling force, synapse onto spinal interneurons and motoneurons to generate stretch reflexes, reciprocal inhibition, and autogenic inhibition that together regulate muscle tone and reflex gain (Oliver et al., 2021). After SCI, disrupted supraspinal control of these reflex arcs, together with altered recruitment of Ia inhibitory interneuron and Renshaw cell circuits, contributes directly to abnormal reflex gain, spasticity, hyperreflexia, and disordered co-contraction (Trompetto et al., 2014; Ko, 2026). Restoring appropriate interneuron subtype identity within reflex pathways is therefore likely to be important for normalizing both voluntary motor control and reflex physiology. Collectively, these excitatory, inhibitory, and commissural motifs embody the fundamental logic of spinal circuit organization, wherein developmental identity dictates intrinsic excitability and connectivity to generate patterned locomotor and autonomic output. This developmental framework also delineates the boundaries of regeneration after injury by linking each subtype’s embryonic specification to its selective vulnerability, circuit role, and therapeutic relevance after SCI (Zholudeva et al., 2021). Excitotoxic and inflammatory cascades preferentially eliminate inhibitory and commissural interneurons, while chronic gliosis and extracellular matrix remodeling degrade residual connectivity and conduction fidelity (Kumru et al., 2010; Hughes and Todd, 2020). The resulting imbalance in inhibitory and excitatory drive manifests as spasticity, hyperreflexia, and neuropathic pain, while loss of commissural coordination abolishes rhythmic stepping (Courtine et al., 2009). Such deficits reflect a breakdown of interneuron physiological function and network coordination rather than mere neuronal loss.

An important conceptual refinement for the PIPM concerns the substantial heterogeneity within progenitor domains. The developmental domains V0–V3 and dI1–dI6 are organizational scaffolds rather than homogeneous functional entities (Bikoff et al., 2016). Within the V1 lineage, for instance, Ia inhibitory interneurons mediating reciprocal inhibition and Renshaw cells providing recurrent inhibition to motoneurons arise from the same *En1*-expressing progenitor pool yet serve functionally distinct circuit roles; comprehensive transcriptomic analyses reveal multiple molecularly distinct V1 subpopulations (Gosgnach, 2023). V3 interneurons, while broadly excitatory and commissural, organize into spatially distinct premotor modules within the lumbar cord, with some receiving direct recurrent motoneuron excitation and others not (Chopek et al., 2018). Interneuron organization also varies along rostrocaudal and mediolateral axes of the spinal cord—a dimension of direct clinical relevance because lesion level and lateral extent differentially disrupt specific circuit subpopulations (Kiehn, 2016; Toossi et al., 2021). Within the PIPM framework, patient-specific regenerative trajectories should therefore account not only for which progenitor domains are recruited, but for which functional subpopulations within those domains are generated, survive, and integrate appropriately given the injury context and residual microenvironment.

## 3 Injury-induced circuit dysfunction

Following SCI, the coordinated function of spinal networks is disrupted as rhythmogenic modules lose excitatory–inhibitory balance and cross-segmental synchrony (Courtine et al., 2009; Kumru et al., 2010). Disconnection of descending supraspinal input destabilizes interneuron ensembles, producing aberrant firing, spastic reflex loops, and fragmented locomotor output (Deska-Gauthier and Zhang, 2019). Within the lesion and spared tissue, surviving interneurons attempt limited reorganization through collateral sprouting, dendritic remodeling, and partial re-engagement of local feedback circuits (Flynn et al., 2011; Zavvarian et al., 2020). Electrophysiological mapping shows that ventral interneurons can reroute sensory input and transiently reconstitute rhythmic activity, but these emergent patterns remain unstable without supraspinal coordination (Harkema et al., 2011; Balbinot et al., 2023). Rehabilitative and sensory-evoked activity transiently restore excitability within dormant CPG nodes, yet the recovered dynamics lack phase precision and may fade without continued stimulation (Duysens and Forner-Cordero, 2019; Singh et al., 2023).

Neuromodulatory approaches can re-entrain surviving circuits by modulating spinal excitability. Epidural spinal cord stimulation delivers patterned electrical pulses through implanted epidural electrodes to engage dorsal root and dorsal column afferent pathways and spinal networks, while non-invasive transcutaneous spinal stimulation recruits overlapping sensory-root and spinal networks to modulate excitability without surgical implantation (Golowasch, 2019; Jervis Rademeyer et al., 2021). Transcranial magnetic stimulation (TMS) represents a distinct corticospinal neuromodulatory approach that may support descending pathway engagement in incomplete injury, rather than constituting a form of direct spinal stimulation, and should be considered separately from spinal stimulation modalities (Fan S. et al., 2025). In parallel, targeted activation of specific interneuron subclasses (V0, V1, V2) or transplantation of stem-cell-derived V2a/V1 populations may partially rebalance excitation and inhibition (Ljunggren et al., 2014; Falgairolle and O’Donovan, 2021). Task-specific rehabilitation, locomotor training, and sensory feedback-guided therapy provide the activity-dependent context required to shape spinal circuit plasticity and must be integrated with any stimulation or regenerative strategy (Wagner et al., 2018). Collectively, these observations reveal that recovery depends less on neuronal replacement than on reinstating coherent interneuron circuit coordination and excitatory–inhibitory balance, highlighting that the core deficit of SCI is one of network disorganization rather than simple neuronal loss.

A critical consideration within precision regenerative frameworks that integrate patient-specific biological state, such as the PIPM, is the distinction between adaptive and maladaptive plasticity. Interventions that amplify excitatory drive to re-engage dormant CPG modules may further shift excitatory–inhibitory balance toward hyperexcitability, spasticity, or autonomic dysreflexia over longer time horizons (Dietz et al., 2022; O’Reilly et al., 2025). Conversely, strategies that strengthen inhibitory networks to manage spasticity may dampen the rhythmogenic excitability required for locomotor re-engagement (Chia and Lin, 2026). The model described here, PIPM, therefore treats plasticity as bidirectional: therapeutic targets must be evaluated not only for immediate functional gains but also for their potential to entrench maladaptive circuit configurations. Some interventions may produce limited immediate improvements in motor output yet preserve ongoing plasticity without negatively interfering with residual circuit reorganization—a trade-off that must be weighed explicitly within any treatment strategy. Longitudinal electrophysiological monitoring, including H-reflex recovery curves, reflex modulation indices, electromyographic (EMG) coherence, gait periodicity, and autonomic variability, may provide candidate physiological readouts for detecting maladaptive shifts before they become stabilized within chronic circuit states (Wagner et al., 2018).

Beneath this circuit-level disruption lies a progressive erosion of the transcriptional programs sustaining interneuron identity and adaptive capacity (Briscoe and Ericson, 1999; Jessell, 2000). Progenitor competence declines as aging, metabolic tone, and inflammation suppress Notch–EGF, SHH, and Wnt signaling, reducing stemness factors such as *SOX2*, *NES*, and *MKI67* (Navarro Negredo et al., 2020). Attenuated SHH/Wnt activity and loss of BMP antagonism diminish GLI1–*β*-catenin signaling and downstream factors (*Chx10*, *Evx1*, *Lhx3*), shifting progenitors toward dorsalized or gliogenic fates and destabilizing CPG architecture (Ribes and Briscoe, 2009). These molecular deficits compress the regenerative bandwidth of the spinal cord by coupling transcriptional fatigue with reduced morphogen sensitivity (Vanacore et al., 2024; Jagadeesan et al., 2025b). Consequently, regenerative outcomes vary widely across individuals, driven by differences in progenitor competence, molecular accessibility, and circuit reintegration capacity (Eavri et al., 2018; Mayhew et al., 2020). This heterogeneity highlights that spinal repair follows no uniform biological rule but instead arises from patient-specific configurations of cellular, molecular, and functional parameters (Jagadeesan et al., 2025a).

## 4 Patient-specific interneuron precision model (PIPM) linking molecular state to circuit recovery

**Figure 2:**
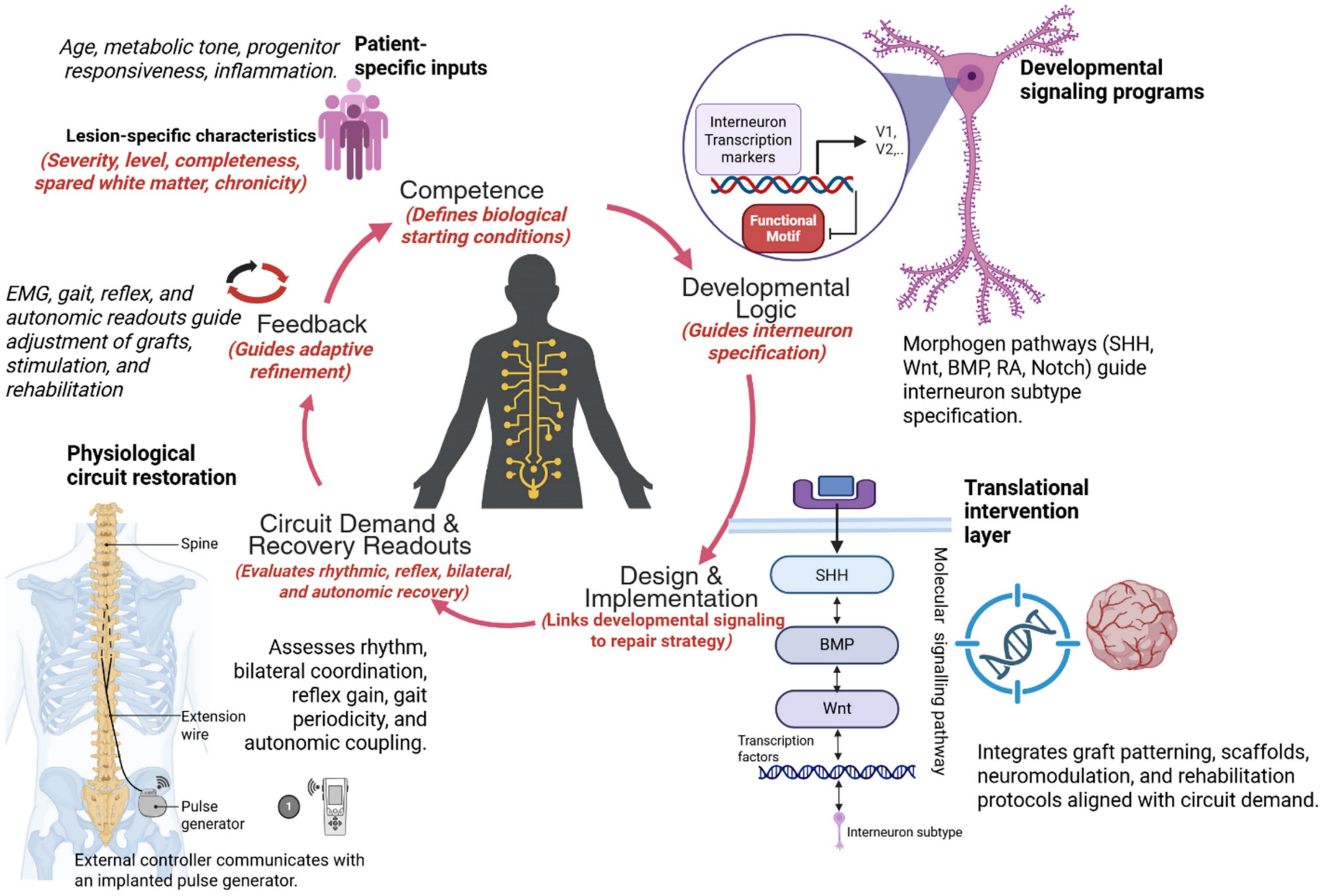
The patient-specific interneuron precision model (PIPM): an adaptive conceptual framework linking biological inputs, developmental logic, design and implementation, and circuit feedback for spinal cord regeneration. The PIPM illustrates how patient-specific biological competence and lesion-specific circuit preservation may guide interneuron-targeted repair strategies. The model progresses through four interconnected domains: Competence, developmental logic, design and implementation, and circuit demand with feedback. Competence defines regenerative starting conditions, integrating progenitor responsiveness, metabolic tone, inflammatory state, lesion severity, spared white matter, and chronicity. Developmental logic represents morphogen-guided interneuron specification through SHH, Wnt, BMP, Notch, and retinoic acid pathways. Design and implementation links developmental signaling to graft patterning, rehabilitation protocols, and neuromodulation strategies aligned with CPG organization. Circuit demand and feedback evaluates rhythmic, bilateral, reflex, and autonomic recovery using EMG, gait, reflex, and physiological readouts to recalibrate intervention parameters.

The PIPM is organized as a four-domain architecture in which each domain corresponds to a distinct stage of the regenerative process (Figure 2). The first domain, competence, characterizes the patient’s intrinsic biological starting conditions: the capacity of NSPCs to decode morphogenetic signals and generate functional interneuron subtypes, defined by morphogen sensitivity, redox equilibrium, metabolic tone, and inflammatory load. The second domain, developmental logic, reactivates the patterning programs that originally constructed the spinal cord, translating competence state into interneuron subtype specification through SHH, BMP, Wnt, Notch, and retinoic acid gradients. The third domain, design and implementation, bridges developmental signaling to circuit reconstruction through engineered grafts, patterned scaffolds, and neuromodulatory strategies aligned with CPG organization. The fourth domain, circuit demand and Feedback, evaluates functional recovery using candidate physiological readouts and uses these as error signals to recalibrate the upstream domains iteratively. Critically, lesion severity, neurological level, injury completeness, spared white matter, chronicity, and residual descending or propriospinal connectivity are primary determinants of recovery potential that may override or substantially modify competence-class predictions; the four domains must therefore be applied together as an integrated framework in which systemic biological state and lesion-specific circuit preservation are evaluated in parallel.

**Figure 3:**
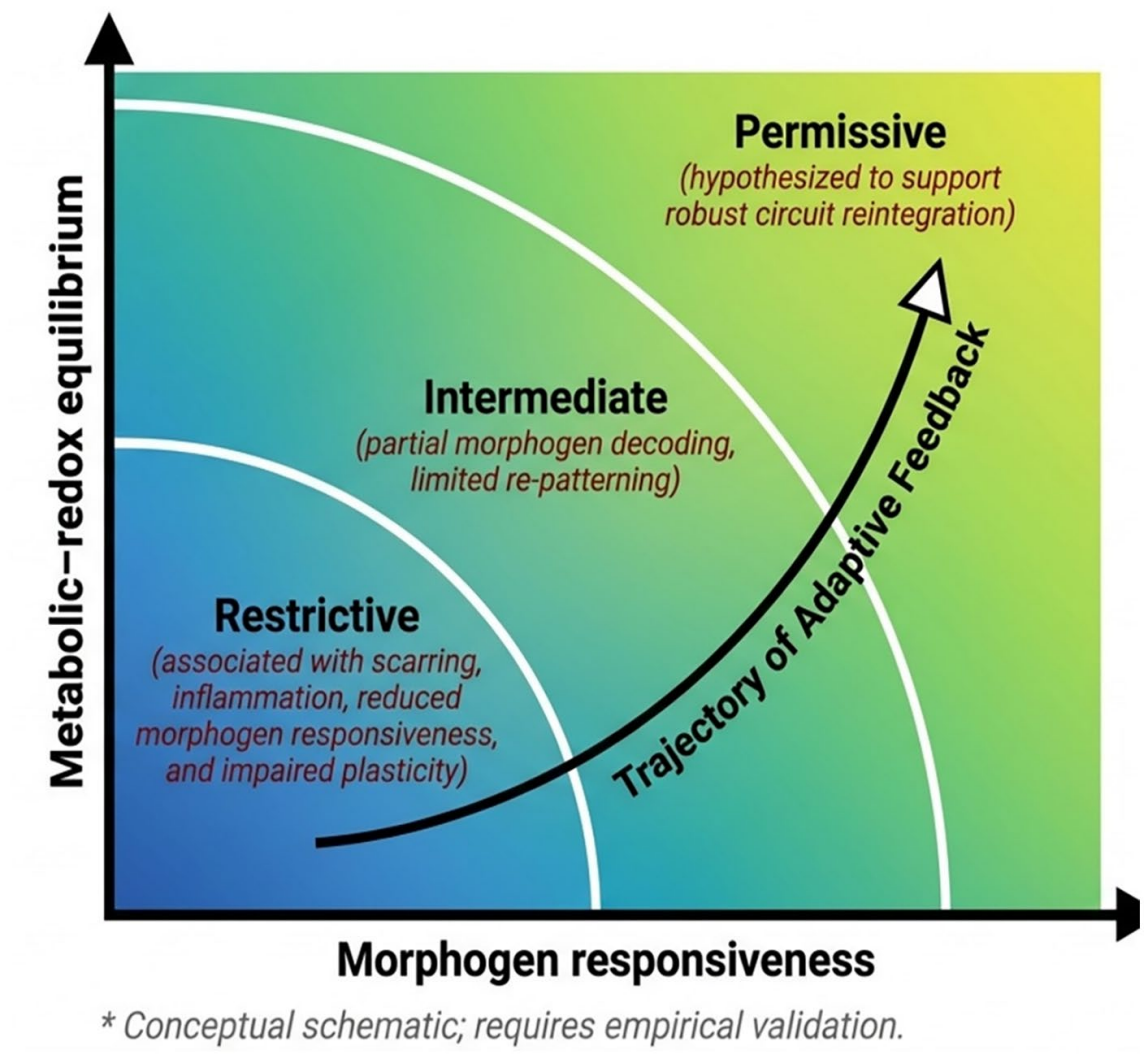
Conceptual competence landscape in the proposed PIPM framework. This schematic represents regenerative potential as a conceptual function of morphogen responsiveness and metabolic–redox equilibrium. Patient-specific biological and injury-related variables may position an individual within a permissive, intermediate, or restrictive recovery landscape. Higher-competence regions are hypothesized to support more effective circuit reintegration, whereas low-competence regions are associated with scarring, inflammation, reduced morphogen responsiveness, and impaired plasticity. This framework is conceptual and requires empirical validation in human-derived experimental systems.

Within the competence domain, the biological state is not a fixed trait but a multidimensional variable that shifts along gradients of morphogen sensitivity, redox equilibrium, metabolic tone, and inflammatory load. High competence reflects efficient morphogen decoding, balanced oxidative metabolism, and accessible chromatin architecture, all of which may sustain transcriptional programs required for neuronal diversification and integration (Smith et al., 2018). Diminished competence, by contrast, may reflect energy deficit, oxidative stress, and pro-inflammatory load, producing restricted differentiation and network fragmentation (Szegedi et al., 2024). Patient-specific metrics may shift this balance and thereby could reshape the regenerative potential of the spinal cord, as conceptually represented across permissive, intermediate, and restrictive recovery landscapes (Figure 3).

Within the developmental logic domain, morphogen gradients of SHH, BMP, Wnt, Notch, and retinoic acid are decoded through transcriptional networks to generate canonical interneuron subtypes-V0 to V3 and dI1 to dI6, that serve as the functional building blocks of spinal circuitry. These subtypes encode distinct rhythmic and regulatory functions. V0 commissural neurons may coordinate left–right alternation, V1 interneurons control flexor–extensor phasing, V2a and V2b units balance excitatory–inhibitory tone, and V3 neurons could sustain bilateral rhythmicity (Deska-Gauthier and Zhang, 2019; Gosgnach, 2023). The success of regeneration therefore may lie not in cellular yield alone but in the accurate reinstatement of these functional motifs that restore feedback gain and temporal coherence within locomotor and autonomic circuits. It should be noted that deterministic predictions, for example, that low competence necessarily produces specific inhibitory subclasses or that high competence will reliably restore function—remain speculative and require empirical validation. Within the circuit demand and feedback domain, the spinal cord is treated as a network of interconnected oscillatory circuits whose healthy dynamics are characterized by stable rhythm, phase synchrony, and intersegmental coordination (Kiehn, 2016; Wenger et al., 2016). Injury displaces these dynamics into desynchronized states, producing fragmented circuit output and unstable motor patterns (Kumru et al., 2010). Functional recovery would require progressive realignment of network dynamics toward stable physiological states characteristic of intact circuitry (Rybak et al., 2015; Guaraldi et al., 2022). Observable metrics such as EMG coherence, reflex modulation indices, gait periodicity, and autonomic variability, may represent candidate physiological readouts for monitoring circuit recovery (Formento et al., 2018; Wagner et al., 2018). These readouts could inform iterative refinement of intervention parameters, including stimulation design, rehabilitation protocols, and, where applicable, graft composition, to better align therapeutic strategies with observed recovery trajectories. Engineered grafts, patterned scaffolds, and closed-loop neuromodulatory interfaces represent potential intervention layers through which such adjustments could be implemented (Griffin and Bradke, 2020; Lorach et al., 2023). Through iterative refinements guided by physiological feedback, spinal networks may progressively transition from disorganized, low-plasticity states toward more coherent circuit configurations supportive of rhythmic motor output.

## 5 Clinical translation

**Figure 4:**
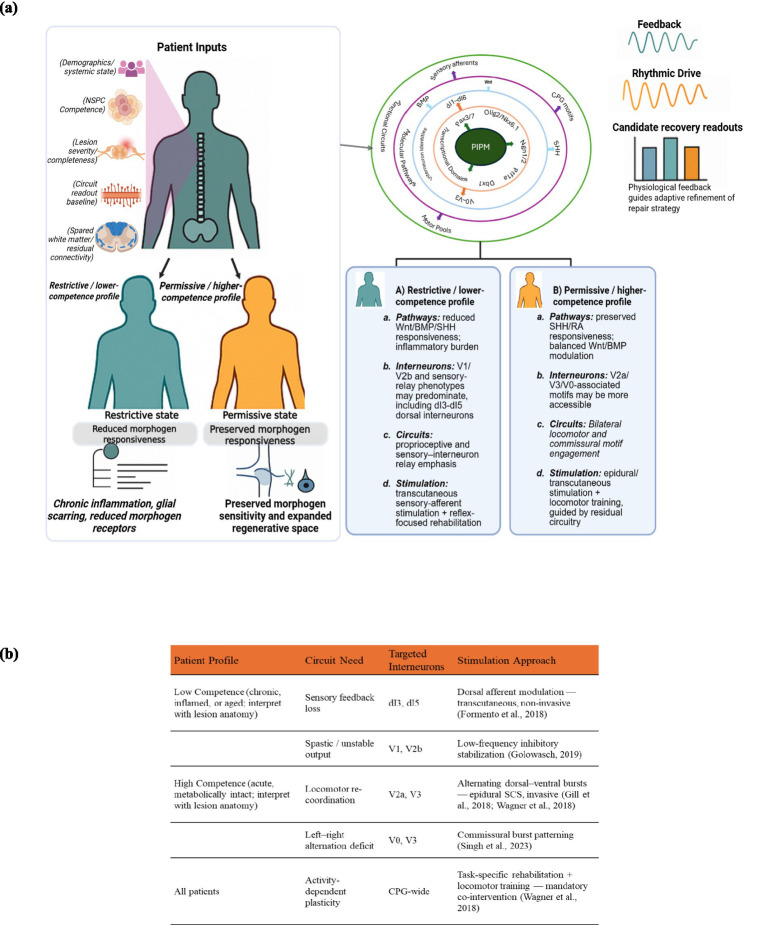
Clinical translation of the patient-specific interneuron precision model (PIPM). **(a)** Low-competence conditions are characterized by attenuated SHH/BMP signaling, glial scarring, and reduced morphogen responsiveness, hypothesized to favor limited inhibitory or sensory-relay interneuron programs. High-competence conditions may expand the regenerative space toward excitatory (V2a, V3) and commissural (V0) populations supporting bilateral rhythm and phase symmetry. Competence-state interpretations must be considered together with lesion level, neurological completeness, spared white matter, and chronicity, which are primary determinants of recovery potential. **(b)** Conceptual circuit-targeted intervention strategies stratified by competence state, linking patient-specific circuit needs to targeted interneuron populations and neuromodulatory approaches. Stimulation modalities include epidural spinal cord stimulation (invasive; Gill et al., 2018), transcutaneous spinal stimulation (non-invasive; Jervis Rademeyer et al., 2021), and TMS (corticospinal; Fan X. et al., 2025). Task-specific rehabilitation must be integrated as a mandatory co-intervention across all competence states (Wagner et al., 2018). All parameters are conceptual illustrations and do not constitute validated clinical prescriptions.

For clinical translation, biological competence cannot be interpreted independently of injury anatomy. Lesion severity, neurological level, completeness, spared white matter, chronicity, and residual descending or propriospinal connectivity are primary determinants of recovery potential and may override competence-class predictions. The PIPM should therefore be applied as an integrated framework in which systemic biological state and lesion-specific circuit preservation are evaluated together (Figure 4a). Within a low-competence regime, often associated with aging, metabolic burden, persistent inflammation, or chronic injury, impaired responsiveness to morphogens coincides with scarring and redox imbalance (Gill et al., 2018). Under these conditions, regenerative programs may preferentially support limited inhibitory or sensory-relay phenotypes rather than the balanced excitatory, inhibitory, and commissural populations required for coordinated rhythmic output, resulting in reduced phase coordination and limited bilateral symmetry (Deska-Gauthier and Zhang, 2019). When metabolic tone and redox control are restored, the effective morphogen window may widen and the probability space for *Chx10*^+^ V2a and *Evx1*^+^ V0 lineages could increase, which is hypothesized to support the re-emergence of locomotor symmetry and more reliable rhythmic drive (Ampatzis et al., 2014; Gosgnach, 2023). These relationships remain conceptual and require prospective validation.

In a high-competence regime, morphogen decoding may remain more intact and progenitor responsiveness could be higher, which may expand the accessible regenerative space (Eavri et al., 2018). Higher-competence conditions might be more permissive for generating or engaging excitatory and commissural populations, including V2a, V3, and V0-associated circuit motifs, potentially supporting more reliable alternation and stronger CPG dynamics (Zavvarian et al., 2020). Recovery may follow from restoring the proportion and timing of inhibitory and excitatory activity rather than from amplifying either alone (Chopek et al., 2018). Iterative adjustment of morphogen exposure, graft composition, and stimulation parameters could improve entrainment and phase stability, with the goal of approaching coherence patterns characteristic of more intact circuitry (Jervis Rademeyer et al., 2021). Across both competence regimes, these considerations suggest that clinical translation of the PIPM is not a single protocol but a stratified decision framework in which biological competence and lesion-specific anatomy are evaluated in parallel to inform interneuron-targeted intervention design. Rather than prescribing fixed therapeutic sequences, the PIPM generates testable hypotheses about which circuit populations are most accessible given a patient’s biological state, and which neuromodulatory or regenerative strategies are most likely to engage them. Prospective validation through human-derived experimental platforms and carefully designed clinical cohorts will be essential to determine whether competence-class stratification can meaningfully predict recovery trajectories and guide precision-guided spinal repair. Conceptual examples of circuit-targeted intervention strategies stratified by competence state, including neuromodulatory approaches and rehabilitation, are summarized in Figure 4b.

## 6 Conclusion

The convergence of developmental biology, patient-specific modeling, and circuit neuroscience is gradually redefining SCI treatment from generalized repair to precision-guided regeneration (Shevtsova et al., 2015; Jagadeesan et al., 2025a). Integrated platforms such as the PIPM embody this transition by viewing spinal repair as the restoration of coordinated physiological circuit function rather than structural replacement alone. The PIPM extends existing frameworks by linking molecular state to circuit-level performance and embedding individual variability directly into regenerative logic. Rather than replacing prior models, it complements them by offering a mechanism to integrate patient data, developmental mechanisms, and physiological feedback into a coherent design framework. Recent advances now make it feasible to evaluate these hypotheses in controlled human-derived systems. Human spinal organoids, assembloids, and microphysiological platforms reproduce segmental identity and rhythmic network behavior, enabling quantitative readouts of progenitor competence and responsiveness (Buchner et al., 2023; Zhou et al., 2024). When combined with multi-omics profiling, electrophysiological mapping, and integrative modeling, these platforms may help estimate recovery trajectories and inform graft patterning or stimulation design (Peng et al., 2024; Rasoolinejad et al., 2025). Integration with bioelectronic interfaces and wearable sensors further supports adaptive, feedback-driven modulation of therapy *in vivo* (Giagiozis et al., 2025).

Key challenges remain, including variability among induced pluripotent stem cell lines, incomplete vascular and biomechanical realism *in vitro*, and the need for rigorous translational and regulatory frameworks for closed-loop neuromodulation (Bikoff et al., 2016; Fan X. et al., 2025). Even so, patient-specific conceptual models such as the PIPM offer a pragmatic step toward individualized regenerative strategies that align therapeutic design with measurable biological competence. Patient variability becomes a parameter to harness rather than a limitation to overcome, guiding the development of more coherent and reproducible recovery outcomes. The PIPM remains a hypothesis-generating conceptual framework that is inherently personalized in its architecture, each domain is parameterized by patient-specific biological and lesion variables, such that the regenerative hypotheses it generates are stratified by individual competence state rather than derived from population-level assumptions. Prospective validation in human-derived experimental models, electrophysiological systems, and ultimately clinical datasets is required before its proposed relationships can be used to inform therapeutic decisions. The framework is intended to stimulate hypothesis-driven research into the molecular, circuit-level, and clinical determinants of recovery, and to provide a foundation for designing controlled studies that can test its core assumptions.

## Data Availability

The original contributions presented in the study are included in the article/supplementary material, further inquiries can be directed to the corresponding author.
